# Effect of low vs. high vancomycin trough level on the clinical outcomes of adult patients with sepsis or gram-positive bacterial infections: a systematic review and meta-analysis

**DOI:** 10.1186/s12879-024-09927-4

**Published:** 2024-10-07

**Authors:** Subhash Chander, Roopa Kumari, Hong Yu Wang, Yaqub Nadeem Mohammed, Om Parkash, Sindhu Lohana, FNU Sorath, Abhi Chand Lohana, FNU Sadarat, Sheena Shiwlani

**Affiliations:** 1 Department of Medicine, Icahn School of Medicine, Mount Sinai, New York, NY USA; 2 Department of Medicine, Montefiore Medical Center, Bronx, NY USA; 3 Department of Medicine, AGA khan University Hospital, Karachi, Pakistan; 4 Department of Medicine, Dow University Health Sciences, Karachi, Pakistan; 5 Department of Medicine, Western Michigan University, Kalamazoo, WV USA; 6 Department of Medicine, University at Buffalo, Buffalo, NY USA; 7 Department of Pathology, Icahn School of Medicine, Mount Sinai, New York, NY USA; 8 Mount Sinai Beth Israel Hospital, 281 1st Ave, New York, NY 10003 USA

**Keywords:** Sepsis, Septic shock patients, Vancomycin, Trough levels, MRSA

## Abstract

**Background & objective:**

The Infectious Disease Society of America guidelines recommend vancomycin trough levels of 15–20 mg/L for severe methicillin-resistant Staphylococcus aureus. However, recent consensus guidelines of four infectious disease organizations no longer recommend vancomycin dosing using minimum serum trough concentrations. Therefore, this study aimed to evaluate the impact of low (< 15 mg/L) vs. high (≥ 15 mg/L) vancomycin trough levels on clinical outcomes in adult patients with sepsis or gram-positive bacterial infections.

**Method:**

A systematic literature review from inception to December 2022 was conducted using four online databases, followed by a meta-analysis. The outcomes of interest included clinical response/efficacy, microbial clearance, length of ICU stay, treatment failure, nephrotoxicity, and mortality.

**Results:**

Fourteen cohort studies met the inclusion criteria from which vancomycin trough concentration data were available for 5,228 participants. Our analysis found no association between vancomycin trough levels and clinical response [OR = 1.06 (95%CI 0.41–2.72], *p* = 0.91], microbial clearance [OR = 0.47 (95% CI 0.23–0.96), *p* = 0.04], ICU length of stay [MD=-1.01 (95%CI -5.73–3.71), *p* = 0.68], or nephrotoxicity [OR = 0.57 (95% CI 0.31–1.06), *p* = 0.07]. However, lower trough levels were significantly associated with reduced microbial clearance [OR = 0.47 (95% CI 0.23–0.96), *p* = 0.04]. Additionally, low trough levels showed a non-significant trend toward a lower risk of treatment failure [OR = 0.89 (95% CI 0.73–1.10), *p* = 0.28] but were significantly associated with a reduced risk of all-cause mortality [OR = 0.74 (95% CI 0.62–0.90), *p* = 0.002].

**Conclusion:**

Except for a lower risk of treatment failure and all-cause mortality at low vancomycin trough levels, this meta-analysis found no significant association between vancomycin trough levels and clinical outcomes in adult patients with sepsis or gram-positive bacterial infections.

## Introduction

Despite a decline in incidence since 1990, an estimated 48.9 million cases of sepsis and 11 million sepsis-related deaths were reported worldwide in 2017 [1]. Even in low-burden countries such as the United States [1], sepsis accounts for 15.6% of deaths among all hospitalized patients [2], while it is the immediate cause of death in about a third of patients admitted to acute care hospitals [3]. Furthermore, 40% of patients with sepsis have a microbiologically documented infection [4]. The most common causative organism in adults with severe sepsis includes gram-negative bacteria such as *Klebsiella pneumoniae* (13.1–19.8%) and *Escherichia coli* (11.7–37.3%) and gram-positive bacteria such as *Staphylococcus aureus* (8.2–14.1%) [5, 6]. Among those with *Staphylococcus aureus* infection, 42% are culture-positive for methicillin-resistant *Staphylococcus aureus* (MRSA) [5], which is highly associated with significant mortality and morbidity due to limited treatment options [7–10].

Vancomycin is the gold standard treatment for MRSA infections, with the highest cumulative clinical experience for various invasive clinical syndromes, including endocarditis, bacteremia, osteomyelitis, and pneumonia [11, 12]. However, its efficacy is currently questioned and criticized owing to its slow bactericidal activity, emerging resistant strains, and serious adverse effects such as hypersensitivity, ototoxicity, and nephrotoxicity [13]. Additionally, several studies have noted a gradual increase in minimal inhibitory concentrations (MICs) of vancomycin against MRSA [14–16], although this finding remains controversial [17–19]. Moreover, there is evidence for altered metabolism, distribution, and elimination of antimicrobial drugs, mainly hydrophilic drugs such as vancomycin, in septic shock or sepsis due to changes in renal clearance and volume of distribution [20].

The AUC/MIC (area under the concentration-time curve to minimum inhibitory concentration) ratio has gained recognition as a more precise predictor of vancomycin efficacy compared to trough levels alone, especially in MRSA infections. Studies have shown that targeting an AUC/MIC ratio of ≥ 400 is associated with improved clinical outcomes, including higher rates of microbial clearance and reduced treatment failure in MRSA infections [21–23]. These findings support the clinical relevance of AUC/MIC-guided vancomycin dosing, aligning with safety data and recommendations outlined in recent guidelines [24]. The 2011 guidelines of the Infectious Diseases Society of America (IDSA) for MRSA infection treatment recommend vancomycin trough concentrations below 15 mg/L for mild infections and 15–20 mg/L for severe infections [12]. The lower trough concentration threshold of 15 mg/L for patients with severe infections is supported by later studies. For instance, a meta-analysis of 4 prospective and 12 retrospective studies by Steinmetz et al. [11] showed that vancomycin concentration below 15 mg/L in patients with severe MRSA infection was associated with higher treatment failure, microbiologic failure, and mortality rates.

However, prolonged therapy and higher serum vancomycin trough concentrations have been associated with nephrotoxicity [11, 25, 26], possibly leading to increased acute kidney injury (AKI), compromising its safety at higher levels [27]. Lodise et al. [28] reported a vancomycin-associated AKI risk of 5% with the initial administration of low trough levels (< 10 mg/L), 21% for moderate (10–15 mg/L) trough levels, 20% for trough levels of 15–20 mg/L, and 33% for trough levels > 20 mg/L which is consistent with findings of other studies [29–31]. Consequently, the most recent consensus guidelines of the American Society of Health-System Pharmacists, the Infectious Diseases Society of America, the Pediatric Infectious Diseases Society, and the Society of Infectious Diseases Pharmacists (2020) no longer recommends vancomycin dosing using minimum serum trough concentrations due to efficacy and nephrotoxicity concerns [32]. Instead, it recommends assuming the vancomycin MIC as 1 mg/L and adopting AUC-guided vancomycin monitoring for MRSA infections [32].

Although MIC/AUC-guided vancomycin dosing and monitoring may be more effective in achieving time to and time in the therapeutic range [33] and possibly a better safety profile, the latest consensus guidelines also underscore the need for caution with this approach while treating mild noninvasive infections and infections caused by non-MRSA species responsive to vancomycin as the guidelines predominantly rely on pharmacological and toxicological data from patients treated for serious for severe MRSA infections [23]. Moreover, recent studies comparing AUC/MIC and trough-only dosing approaches have yielded inconsistent results in terms of safety. For instance, Folkers et al. [33] did not find any significant difference in the incidence of AKI with the two dosing approaches modality, while McClure et al. [34] reported a 23% lower risk of incident AKI with the AUC/MIC-guided approach versus trough-only approach. In addition, the AUC/MIC-guided approach is associated with a marginally higher cost of vancomycin dosing and monitoring [35], which may be limiting in resource-strapped settings or facilities with large caseloads.

Given that the data with that AUC/MIC-guided approach is still emerging and the trough-based approach will continue to be relevant for mild or non-MRSA infection and in limited resource settings, continuously evaluating emerging literature on vancomycin dosing and monitoring is essential. Therefore, this systematic review and meta-analysis aimed to assess the impact of low (< 15 mg/L) vs. high (15–20 mg/L) trough concentration of vancomycin on the clinical response/efficacy, microbial clearance, length of intensive care unit (ICU) stay, treatment failure, nephrotoxicity, and mortality in patients with sepsis or gram-positive bacterial infections including MRSA.

## Methods

This systematic review and meta-analysis conformed to the guidelines provided by the Cochrane Collaboration Search Strategy and Preferred Reporting Items for Systematic Reviews and Meta-Analysis (PRISMA) statement [36, 37].

### Search strategy

Scopus (Medline), PubMed, Cochrane Central Register of Controlled Trials (Central), and Google Scholar databases were searched using a varied mix of search terms that included keywords and MeSH terms (vancomycin, pharmacokinetics, critically ill, ICU, efficacy, safety, AUC/area under the curve, MIC, trough, and AKI). Additionally, the reference lists of all potential articles were screened and manually retrieved for additional articles.

### Inclusion criteria

Our inclusion criteria were guided by the PICOTS framework as follows:

- *Population*: Adult patients (> 18 years) with sepsis (including septic shock as a subset of sepsis) or gram-positive bacterial infections.
- *Intervention*: Vancomycin.
- *Comparison* Low (< 15 mg/L or 10-15 mg/L) vs. (15-20 mg/L or ≥ 15 mg/L) trough levels of vancomycin. We did not include troughs of > 20 in our analysis due to the very high risk of toxicity.
- *Outcomes* Treatment success/failure, clinical response, microbial clearance, mortality, ICU stay, bacterial recurrence, and/or nephrotoxicity.
- *Timing* Since inception to December 2022.
- *Setting & design* Controlled trials (randomized and nonrandomized) and cohort (retrospective and prospective) studies published in English.

### Study screening, selection, and data extraction

Articles identified from the database search were electronically retrieved for screening. Two authors (SC and RK) screened, selected, and extracted data from articles meeting our inclusion criteria. The process involved identifying duplicate entries, title and abstract screening, and full-text screening while removing articles not meeting our inclusion criteria at each step. All disagreements were resolved by discussion and consensus between the two reviewers. However, the two authors independently conducted the data extraction process using a standard data extraction form comprising study setting and location, design, study duration, sample size, age and gender of participants, infection type, defined breakpoint, objectives, and relevant findings.

### Outcomes and definitions

The primary study outcomes were clinical response/efficacy, microbial clearance, ICU length of stay, and all-cause mortality. All-cause mortality was defined as 30-day, in-hospital, or ICU mortality.

Secondary study outcomes included treatment failure and nephrotoxicity. Treatment failure was defined as a composite endpoint including at least one of the following: death from any cause within 30 days of treatment, microbiologic failure/bacterial persistence after seven days of vancomycin therapy, or recurrence of the bacterial infection within 60 days of discontinuing vancomycin therapy.

For the meta-analysis, vancomycin trough levels from the included studies were dichotomized into low, defined by serum trough of < 15 mg/L, and high, defined by serum trough of 15–20 mg/L.

### Risk of bias and study quality assessment

Two reviewers (RK and SC) independently evaluated the risk of bias in the included articles that fulfilled the inclusion criteria. Most studies selected for this review were retrospective and prospective cohorts, so the quality assessment was evaluated based on the Newcastle-Ottawa Scale [38]. The following items were assessed: (a) study selection criteria, including representativeness of the exposed cohorts, selection of the non-exposure group, and ascertaining the exposure levels; (b) comparability of the study groups; and (c) outcomes, including assessment of various clinical outcomes (independent blind assessment/record linkage/self-report/no description), and follow-up duration for outcomes to occur.

### Statistical analysis

All statistical analyses were conducted using R software (Version 2024.04.2 + 764). Odds ratios (ORs) and 95% confidence intervals (CI) were used to calculate the effect sizes of individual studies by producing forest plots. Heterogeneity in the results of the analyzed studies was assessed using the chi-square test for study heterogeneity and the I² statistic to measure inconsistency and heterogeneity degree [39, 40]. If no inter-study heterogeneity was detected, a meta-analysis was conducted using the Mantel-Haenszel fixed-effects model approach. Otherwise, if the studies showed significant heterogeneity, the meta-analysis was performed using the Mantel-Haenszel random-effects model approach. Funnel plots were generated to assess the degree of asymmetry tested by Egger’s [41] and Begg’s test. A *p*-value < 0.05 was considered statistically significant.

## Results

### Literature search and selection results

**Fig. 1:**
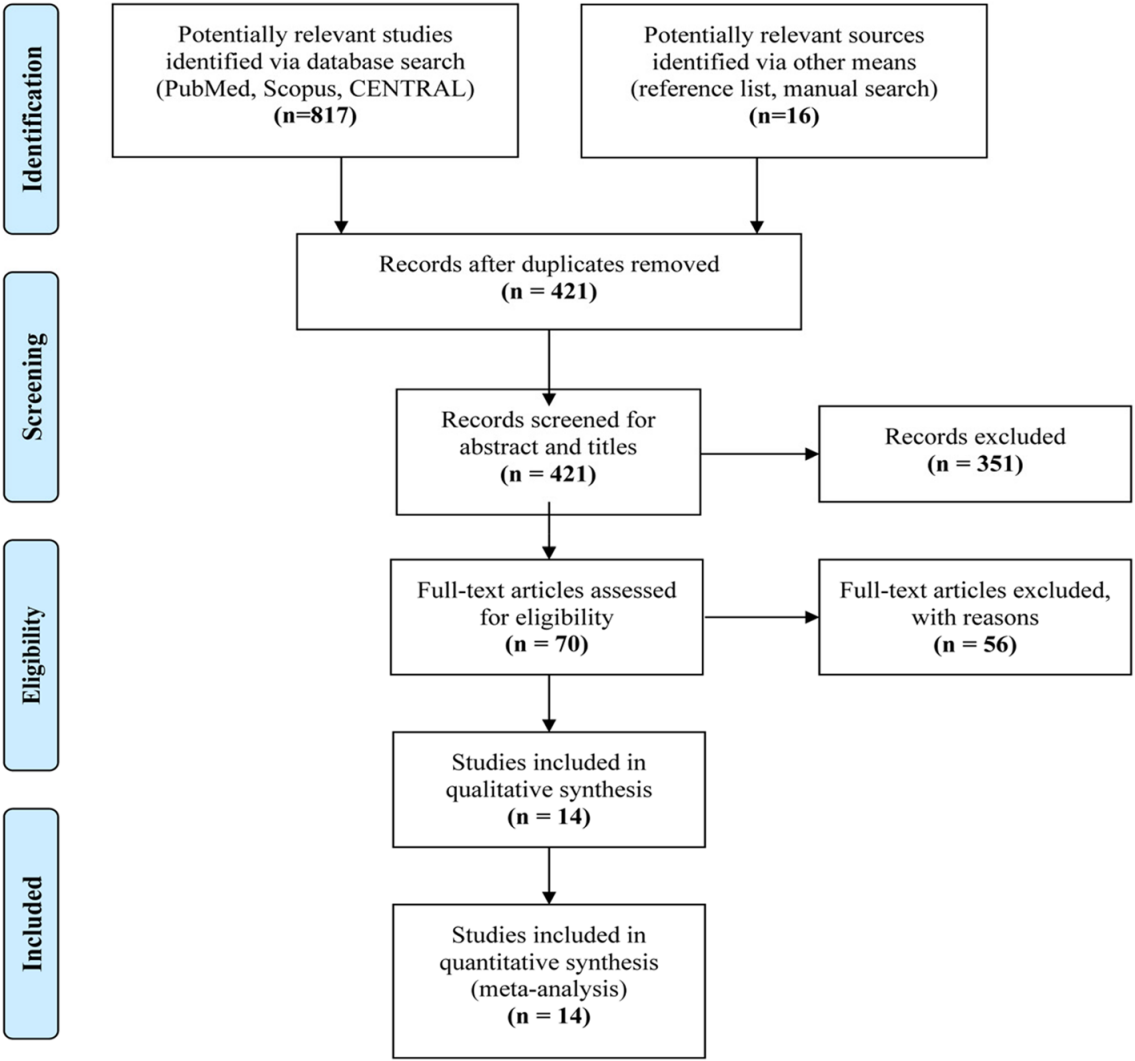
PRISMA Flow Diagram of Study Selection Process

Our search strategy yielded 817 articles from databases and 16 from the reference list screening, of which 412 were eliminated as duplicate studies. The remaining 421 records were screened based on titles and abstracts in conformity with the inclusion criteria, eliminating 356 studies. Seventy studies were eligible for full-text screening, of which 14 articles met the inclusion criteria and were included in this meta-analysis. The study screening and selection process is illustrated in Fig. 1.

### Characteristics of included studies

The characteristics of the 14 included articles are listed in Table 1. Eleven included studies were retrospective observational cohorts [42–52], and three were prospective studies [53–55]; we did not identify any randomized controlled trials meeting our inclusion criteria. Regarding regional distribution, six studies were from the United States [42–46, 53], two from China [47, 48], and each from Korea [54], Japan [49], Israel [50], Iran [51], France [55], and Slovakia [52]. All the included studies were published between 1997 and 2023. Twelve studies included patients with laboratory-documented MRSA infections and two with documented gram-positive infections (including MRSA).

**Table 1:** Summary and characteristics of included studies

Author (Year) Country	Study duration	Study setting	Sample size	Age	Infection type	Outcomes	Key findings
Retrospective cohort studies							
Jeffres et al. [42] (2006) USA	Jan 1999 - Jun 2005	Inpatient including ICU	102	Mean age 59.4 ± 15.3 years	MRSA	Mortality, ICU length of stay	Aggressive dosing strategies for vancomycin (e.g., trough concentrations of > 15 g/mL) may not offer any advantage over traditional dose targets (range, 5 to 15 g/mL).
Hermsen et al. [43] (2010) USA	Jun 2005 - Jun 2007.	Inpatient including ICU	55	Mean age 60 years	MRSA	Clinical response, mortality, length of stay, and nephrotoxicity	-Mortality risk was not significantly different between the high (19%) and low (5%) trough group patients (*p =* 0.1). -LOS did not differ significantly between groups (*p =* 0.7). -Nephrotoxicity occurred in the low and high groups, respectively, for 10% and 31% (*p =* 0.04). -There was no significant difference between high and low trough levels on clinical outcomes for MRSA infections. However, nephrotoxicity was higher in the high trough group.
Kullar et al. [44] (2011) USA	Jan 2005 - Apr 2010	N/R	320	IQR 46–64 years	MRSA	Clinical outcome, treatment failure, and nephrotoxicity.	Vancomycin 10-14.9 mg/l reported more treatment failure (57.8%) and nephrotoxicity (17.1%) than 15–20 mg/l trough levels (39.5%) and nephrotoxicity (13%).
Clemens et al. [45] (2011) USA	Apr 2008 - Aug 2009	Inpatient including ICU	118	Mean age 53 years (range: 18–89)	MRSA	Treatment failure, clinical efficacy	Treatment outcomes were similar regardless of VAN MIC, although there was a non-statistically significant trend towards decreased clinical efficacy among patients with VAN MIC = 2 mg/L. Optimization of VAN pharmacokinetic indices did not appear to correlate with clinical responses.
Hou et al. [46] (2021) USA	2014 to 2015	ICU	3,603	45.6% ≤ and 53.5% > 60 years	MRSA	ICU and hospital mortality	The mean vancomycin trough concentration (VTC) did not influence reduced ICU/ hospital mortalities, thus suggesting that VTC does not guarantee treatment efficacy for ICU patients.
Wang et al. [47] (2021) China	Jan 2017 -Dec 2019	Inpatient	349	Mean age 88 years	Complicated Gram-positive infection	Clinical response, 30-day mortality rates, persistent bacteremia, nephrotoxicity	-For patients with VTCs at < 10, 10–15, 15–20, and ≥ 20 µg/mL, the clinical response rates were, respectively, 77.8, 77.0, 80.5, and 61.0%; the 30-day mortality rates were 2.8, 15.0, 15.3, and 37.8%; and the rates of persistent bacteremia were 16.7, 12.4, 11.9, and 11.0%. -Higher VTC level was not associated with favorable treatment outcomes.
Huang et al. [48] (2018) China	Jan 2007 - Jun 2014	ICU	50	Mean age 85.0 ± 3.9 years	Gram-positive infection	Mortality, clinical outcome, and nephrotoxicity	The 28-day mortality was 26.0% (13/50). Of the patients, 24% (12/50) had nephrotoxicity during the vancomycin treatment. The clinical efficacy was 60%, 86.7%, 58.3%, and 33.3%, and the 28-day mortality rate was 20%, 23.3%, 33.3%, and 33.3%, respectively, when the trough concentrations were ≤ 10 µg/mL, 10–15 µg/mL, 15–20 µg/mL, and ≥ 20 µg/mL.
Chuma et al. [49] (2018) Japan	Between 2005 and 2015	ED and ICU	109	Mean age 67 years	MRSA, Coagulase-negative Staphylococcus spp, Enterococcus spp	Nephrotoxicity	Nephrotoxicity incidence rate was 14.3% in patients with initial trough levels of 15–20 mg/L, higher than 12.5% in patients with initial trough levels of 10 < 20 mg/L.
Yahav et al. [50] (2019) Israel	Jan 2013 – Dec 2015	Inpatient excluding ICU	285 patients	Mean age 67 ± 15.8 years	MRSA	30-day all-cause mortality, clinical success, microbiological success, or nephrotoxicity	-There were no significant differences between patients achieving high and low vancomycin levels in mortality (46/131, 35.1% vs. 41/154, 26.6%), clinical success, microbiological success, or nephrotoxicity. -The study found no association between vancomycin levels > = 15 mg/L and clinical outcomes in patients with MRSA infection.
Arasteh et al. [51] (2019) Iran	NR	ICU	39	42.18 ± 3.84 for low trough and 48.09 ± 9.54 for high trough patients	MRSA	Clinical response and microbiological clearance	-There was no difference between the groups on clinical response (*p =* 0.677) and microbiological clearance (*p =* 1.00) -Both patients’ groups had comparable outcomes regardless of trough levels of vancomycin.
Kralovicova et al. [52] (1997) Slovakia	Jan 1990 to Dec 1995	Inpatient including ICU	198	NR	MRSA	Treatment failure, nephrotoxicity	A high trough level reported more nephrotoxicity (33.3% vs. 11.1%) than a low trough level.
Prospective cohort studies							
Bosso et al. [53] (2011) USA	Feb 2008 - Jun 2010	Inpatient including ICU	288	Mean age 55 ± 17 years	MRSA	Nephrotoxicity, ICU length of stay	Vancomycin trough concentrations of > 15 mg/ml associated with a 3-fold increased risk of nephrotoxicity and ICU length of stay.
Chung et al. [54] (2011) Korea	Aug 2005 - Jul 2007	ICU	141 (Intention-to-treat analysis of 68 patients with MRSA)	Mean age 62.7 ± 14 years	MRSA	Treatment success rate, length of ICU stays, and ICU mortality rate	No significant differences were observed in the treatment success rate, length of ICU stay, and ICU mortality rate between patients with vancomycin trough concentrations of > 20 mg/l, 15 to 20 mg/l, and < 15 mg/l.
Arshad et al. [55] (2012) France	Jul 2005 - Mar 2007	N/R	104	N/R	MRSA	Clinical response, mortality, and nephrotoxicity	A low trough level reported less nephrotoxicity, mortality, and medication failure than a high trough level.

*ICU *Intensive care unit, *MIC *minimum inhibitory concentration, *MRSA *methicillin-resistant Staphylococcus aureus, *N/R *Not reported

The 14 included studies provided a pool of 5,228 adult participants with vancomycin trough levels: two studies had four categories of vancomycin trough levels (< 10, 10-14.9, 15–20, and > 20 mg/L), three had three categories of trough levels (< 15, 15–20, and > 20 mg/L), and the remaining studies had two trough concentration levels (< 15 and ≥ 15 mg/L). The studies reported varied data for investigating the association between various vancomycin trough levels and clinical and drug resistance outcomes.

### Quality of included studies

Although the methodological quality of the fourteen included articles varied, it did not influence their inclusion for analysis. The scores of studies ranged from 8 to 9 on the Newcastle-Ottawa Scale. None of the articles attained a score of ≤ 7, indicating that the overall quality of the included studies was high. The details of the risk of bias assessment are shown in Table 2.

**Table 2:** Newcastle Ottawa Scale (NOS) for Quality Assessment

Study	Selection			Comparability		Outcome			Total
	Representativeness of the exposed cohort	Selection of the non-exposed cohort	Ascertainment of exposure	Demonstration that outcome of interest was not present at the start of the study	Comparability of cohorts based on the design or analysis	Assessment of outcome	Duration of follow-up enough for outcomes to occur	Adequate follow-up	
Full score	**1**	**1**	**1**	**1**	**2**	**1**	**1**	**1**	**9**
Jeffres et al. [42] (2006)	1	1	1	1	1	1	0	1	7
Hermsen et al. [43] (2010)	1	1	1	1	2	1	0	1	8
Kullar et al. [44] (2011)	1	1	1	1	2	0	1	1	8
Clemens et al. [45] (2011)	1	1	1	1	1	1	1	1	8
Hou et al. [46] (2021)	1	1	1	1	2	1	1	1	9
Wang et al. [47] (2021)	1	1	1	1	2	1	1	1	9
Huang et al. [48] (2018)	1	1	1	1	2	1	0	1	8
Chuma et al. [49] (2018)	1	1	1	1	2	1	0	1	8
Yahav et al. [50] (2019)	1	1	1	1	2	1	1	1	9
Arasteh et al. [51] (2019)	1	1	1	1	2	1	1	1	9
Kralovicova et al. [52] (1997)	1	1	1	1	2	1	0	1	8
Bosso et al. [53] (2011)	1	1	1	1	2	1	1	1	8
Chung et al. [54] (2011)	1	1	1	1	2	1	0	1	8
Arshad et al. [55] (2012)	1	1	1	1	2	1	1	1	9

### Primary outcomes

#### Clinical response/efficacy

**Fig. 2:**
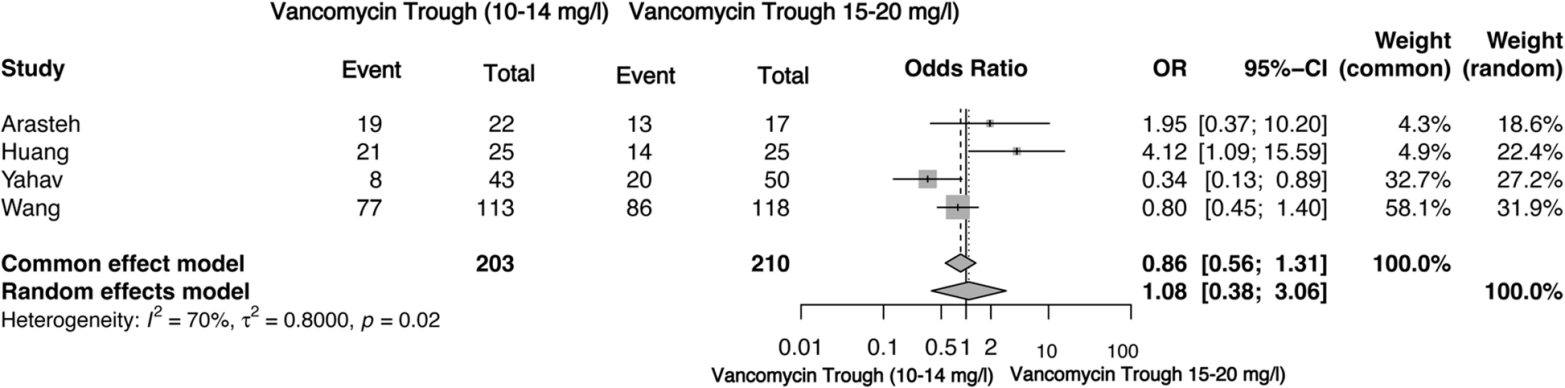
Forest plot of comparison: clinical response/efficacy

Data for patient clinical outcomes were available from four cohort studies [47–51], which collectively provided a study sample of 203 participants with low and 210 with high vancomycin trough levels. A random effect model was used for the meta-analysis since the studies reported significant heterogeneity (I² = 70%). The results showed no significant difference in the clinical response of patients with low or high trough levels of vancomycin [OR = 1.06 (95%CI 0.41–2.72], *p* = 0.91] (Fig. 2). A sensitivity analysis was performed to investigate the source of high heterogeneity detected in this meta-analysis. The exclusion of two studies [47, 50] demonstrated no heterogeneity; hence, it was presumed that the representativeness of the participants, i.e., possible selection bias of participants and ascertainment of the exposure, could confound the high heterogeneity.

#### Microbial clearance

**Fig. 3:**
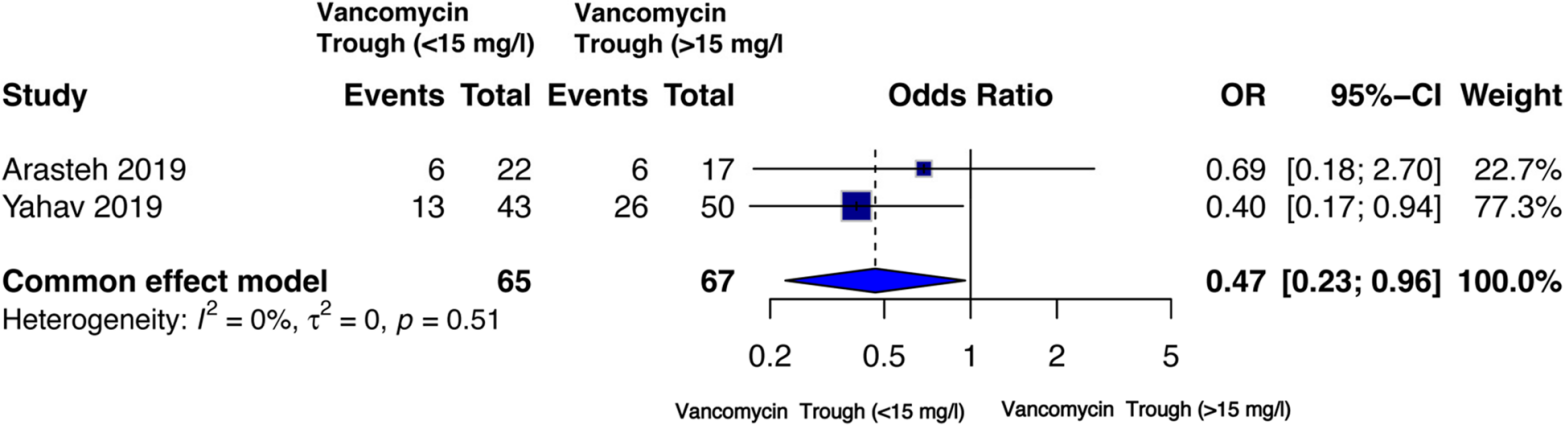
Forest plot of comparison: microbial clearance

Two studies reported microbial clearance with low (*n* = 65) or high (*n* = 67) trough levels of vancomycin [50, 51]. A fixed effect model was used for meta-analysis as the studies had no heterogeneity (I² = 0%). The analysis indicates significantly lower odds of microbial clearance [OR = 0.47 (95% CI 0.23–0.96), *p* = 0.04] with low vancomycin trough concentrations (Fig. 3).

#### ICU length of stay

**Fig. 4:**
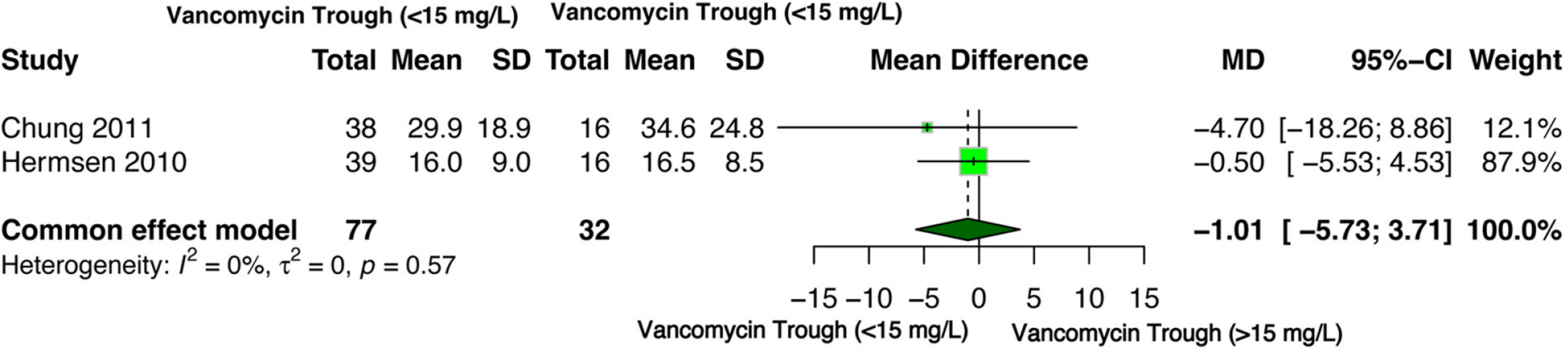
Forest plot of comparison: ICU length of stay

Two studies reported ICU length of stay with low (*n* = 77) or high (*n* = 32) trough levels of vancomycin [43, 54]. A fixed effect model was used for the meta-analysis since the studies reported no heterogeneity (I² = 0%). Trough levels of vancomycin were not associated with ICU length of stay [MD= -1.01 (95%CI -5.73–3.71), *p* = 0.68] (Fig. 4).

#### All-cause mortality

**Fig. 5:**
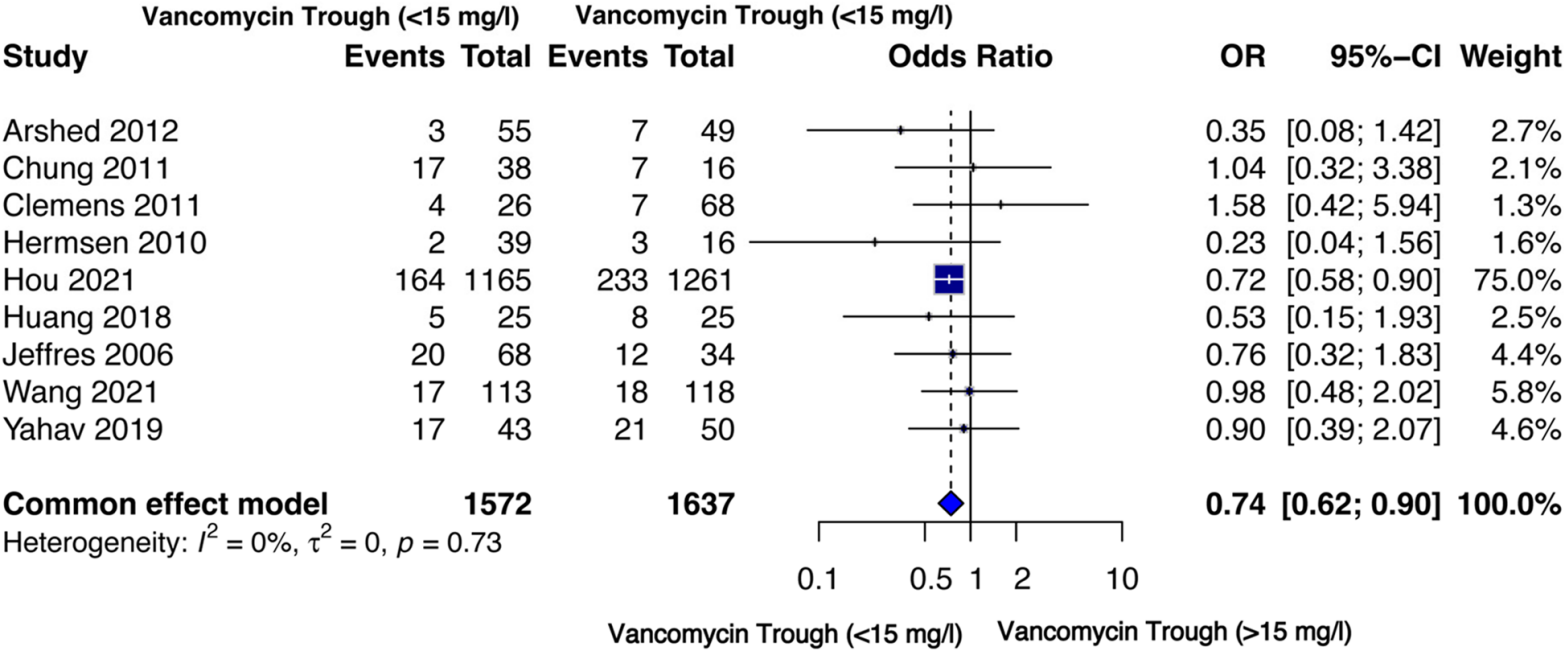
Forest plot comparison: All-cause mortality

Mortality data with low (*n* = 1,572) or high (*n* = 1,637) trough levels of vancomycin were available from 9 studies [42, 43, 45–48, 50, 54, 55] with no inter-study heterogeneity (I² =0%). Low vancomycin trough level was associated with a significantly lower mortality risk in the fixed effect model [OR = 0.74 (95%CI 0.62–0.90], *p* = 0.002] (Fig. 5).

### Secondary outcomes

#### Treatment failure

**Fig. 6:**
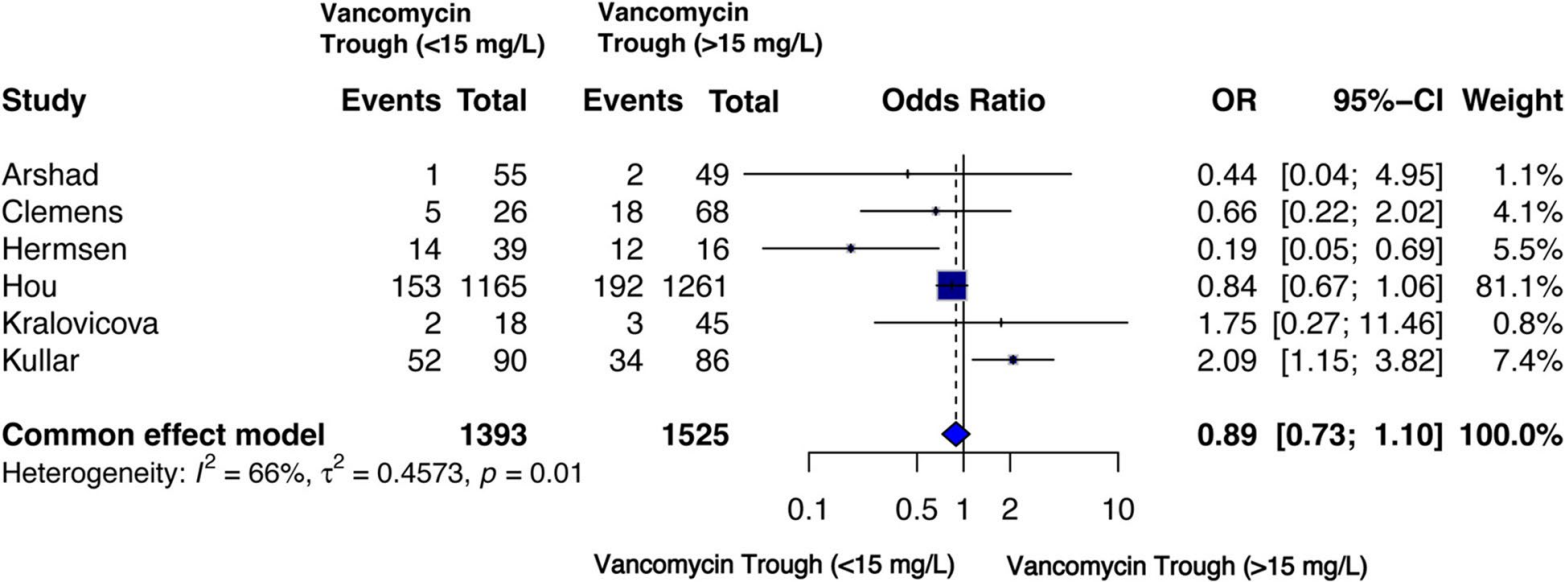
Forest plot of comparison: Treatment failure

Treatment failure was reported in six studies [43–46, 52, 55] with a large sample size of 2,918 patients (n for < 15 mg/L = 1,393 and for ≥ 15 mg/L = 1,525) and significant inter-study heterogeneity (I² = 66%). In the fixed effect model, low vancomycin trough levels were associated with non-significant trend toward a lower risk of treatment failure compared with higher trough levels [OR = 0.89 (95% CI 0.73–1.10), *p* = 0.28] (Fig. 6).

#### Nephrotoxicity

**Fig. 7:**
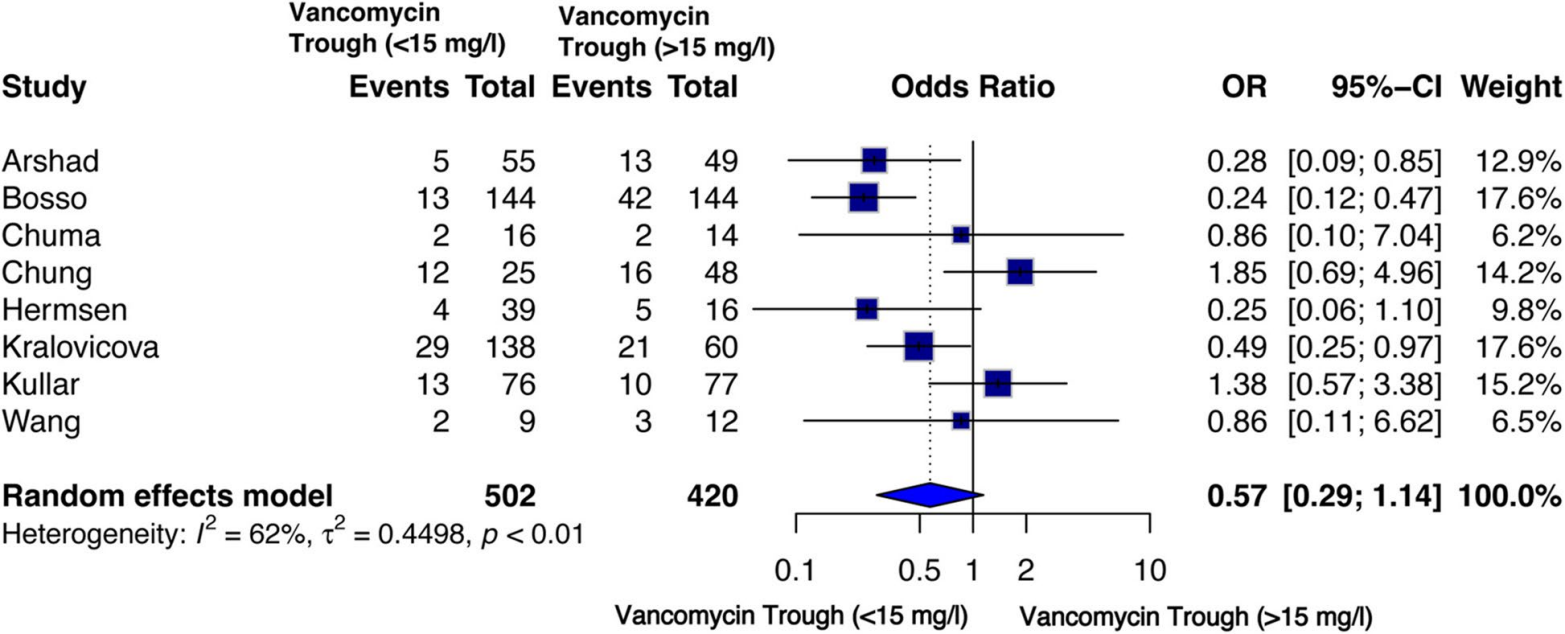
Forest plot of comparison: Nephrotoxicity

Nephrotoxicity was reported in eight studies [43, 44, 47, 49, 52–55], providing a sub-population of 502 patients with low trough and 420 patients with high trough levels of vancomycin with significant inter-study heterogeneity (I² = 62%). In the random effects model, trough levels of vancomycin were not associated with nephrotoxicity [OR = 0.57 (95% CI 0.31–1.06), *p* = 0.07] (Fig. 7). However, due to the significantly high heterogeneity detected, a sensitivity analysis was done to determine the possible cause of the variability. An analysis by excluding three studies [44, 49] showed no heterogeneity among the other studies, implying that the high heterogeneity could be due to the high variability of patient data, i.e., serum creatine levels, receiving first vancomycin therapy a few days before the study and any other possible covariates.

## Discussion

This meta-analysis evaluated the clinical and drug resistance outcomes associated with low and high vancomycin trough levels in adult patients with sepsis or gram-positive bacterial infections, including MRSA. Our analysis found no significant association between vancomycin trough levels and clinical response, microbial clearance, ICU length of stay, or nephrotoxicity. However, low trough levels were associated with a non-significant trend toward a lower risk of treatment failure and a significantly reduced risk of all-cause mortality.

Our findings contradict previous meta-analyses in this domain. For instance, Tongsai et al. [56] reported a higher risk of nephrotoxicity with high vancomycin trough levels while noting a null association between trough levels and clinical success or all-cause mortality. Similarly, Prybylski [29] showed that vancomycin trough levels were not associated with treatment failure, persistent bacteremia, or mortality. Furthermore, Steinmetz et al. [11] found no significant difference between low and high vancomycin trough levels and all-cause mortality or treatment failure rates. However, low and high vancomycin levels were associated with higher microbiologic failure rates and nephrotoxicity, respectively. Finally, Meng et al. [57] reported an increased risk of nephrotoxicity with high vancomycin trough concentrations, although trough concentrations were not associated with the risk of treatment failure and all-cause mortality.

These inconsistencies may be attributable to methodological differences. Our study population comprised patients with sepsis, gram-positive bacterial infection, or MRSA, the three most common indications for the vancomycin regime [13]. In contrast, Tongsai et al. [56] sampled studies reporting nephrotoxicity in patients with MRSA irrespective of infection site, Prybylski [29] specifically sampled patients with MRSA bacteremia, and Meng et al. [57] sampled patients with gram-positive bacterial infections including MRSA. Moreover, Tongsai et al. [56] and Prybylski [29] excluded patients with vancomycin trough levels > 20 mg/L in their meta-analysis. Although Steinmetz et al. [11] sampled patients with MRSA infections and sepsis, they excluded studies that only reported nephrotoxicity without efficacy outcomes.

Nonetheless, these inconsistencies support the notion that vancomycin trough level may not be a reliable predictor of clinical outcomes, in line with the latest consensus guidelines of the American Society of Health-System Pharmacists, the Infectious Diseases Society of America, the Pediatric Infectious Diseases Society, and the Society of Infectious Diseases Pharmacists not to use minimum serum trough concentrations for vancomycin therapeutic drug monitoring (TDM) [32]. Moreover, though monitoring vancomycin trough levels has been suggested to improve efficacy and safety of clinical outcomes and reduce nephrotoxicity and drug failure in gram-positive patients, an effective target trough level is still not defined since the recommendations of 15–20 mg/L trough levels do not guarantee better outcomes.

As previously noted by Chung et al. [54] and Clemens et al. [45], our study indicates that patients would probably exhibit poor clinical success when administering high trough vancomycin levels. Furthermore, we observed a lower risk of treatment failure, defined as bacterial persistence with low trough levels. In agreement with this finding, Prybylski [29], Hale et al. [58] also showed that persistent bacteremia was higher in patients with high vancomycin trough levels.

Although several studies have previously demonstrated vancomycin-induced nephrotoxicity [25, 28, 59, 60], the non-significant reduction in the risk of vancomycin-induced nephrotoxicity at low trough levels observed in the current study warrants further investigation as this could indicate nephrotoxicity even at low trough levels or high variability in nephrotoxicity at high trough levels. In support of the latter, a meta-analysis by van Hal et al. [61] noted that nephrotoxicity incidence rates varied between 7% and 67% in patients exposed to elevated trough levels compared to 0 to 33% in the low serum trough level group. Additionally, Pan et al. [62] recently demonstrated that vancomycin-associated nephrotoxicity was associated with trough concentration ≥ 20 mg/L, and given that most studies included in the current meta-analysis report trough concentration < 20 mg/L, the incidence among low and high trough groups may not be sufficiently large to achieve statistical significance. It is also important to note that although Steinmetz et al. [11] demonstrated a higher risk of nephrotoxicity with vancomycin levels of ≥ 15 mg/L, irreversible renal damage was not reported in any of the cases of vancomycin-induced nephrotoxicity.

### Limitations

Several limitations prevent the generalization of our findings. First, all studies included in this systematic review and meta-analysis were observational cohort studies, most with small sample sizes. These studies may be subject to selection bias for the participant impacting the quality of the study results. Moreover, most of the studies included were conducted in ICU settings, suggesting a higher potential for bias related to overall mortality and renal dysfunction. This bias could stem from vancomycin itself, septic shock, or nephrotoxicity from other medications.

Second, not all studies reported the initial trough value or only reported the average trough value. The successful treatment of MRSA infections could also be confounded by factors unrelated to vancomycin trough levels, such as adequate drainage and appropriate duration of therapy. These are difficult to isolate in this study. Additionally, vancomycin dosing is influenced by renal function and the severity of the disease, indicating that target trough levels may vary among different patient populations. Third, we could not consider vancomycin MIC as 8 of the 14 included studies were from over a decade ago, which did not allow the extraction of the distribution of MIC values. Fourth, there is an inherent risk of publication bias since positive studies were more likely to be published than negative ones. Fifth, targeted analysis was not possible due to the presence of confounding factors, limiting the ability of this study to establish a definite causal association between the study variables.

Finally, baseline patient characteristics such as disease severity and underlying comorbidities may have influenced our results. For instance, among the six studies included in our meta-analysis for treatment failure, three reported significantly high Acute Physiology and Chronic Health Evaluation (APACHE) scores among patients in ≥ 15 mg/L trough group [43, 46, 55], and one reported higher prevalence of heart failure and ICU admission [45] indicative of severe clinical status at baseline. APACHE score was not significantly different between the two trough groups in Kullar et al. [44], while Kralovicova et al. [52] did not report baseline clinical characteristics of the study population. Similarly, three out of nine studies included in our meta-analysis for all-cause mortality reported higher APACHE scores in the ≥ 15 mg/L trough group [43, 46, 55], one reported higher prevalence of heart failure and ICU admission [45], while two reported non-significant differences in APACHE scores [42, 54] and one reported non-significant differences in Charlson comorbidity index [50] between the two trough groups. Two studies did not report baseline APACHE scores for different trough groups [47, 48]. Incidentally, the four studies that reported a more severe clinical status at baseline in the ≥ 15 mg/L trough group contributed 92% of our pooled study population (and 81% of events) for treatment failure and 83% of our pooled study population (and 75% of events) for all-cause mortality. It is crucial to note that including infections caused by pathogens other than MRSA, such as enterococci and Streptococcus, in the current study may add additional variability in disease severity and outcomes.

Nevertheless, the current study provides a comprehensive analysis of the impact of low and high vancomycin trough levels on clinical outcomes, including two studies since the publication of the consensus guidelines of the American Society of Health-System Pharmacists, the Infectious Diseases Society of America, the Pediatric Infectious Diseases Society, and the Society of Infectious Diseases Pharmacists recommending MIC/AUC-guided vancomycin dosing and monitoring for MRSA infections [32]. Of these, the study by Hou et al. [46] is one of the biggest to date in terms of participant size (*n* = 3,603) to report the association between vancomycin trough levels and mortality.

## Conclusion

With the exception of a non-significant trend toward a lower risk of treatment failure and a significant reduction in all-cause mortality at low vancomycin trough levels, this meta-analysis did not detect any significant association between vancomycin trough levels and clinical outcomes in adult patients with sepsis or gram-positive bacterial infections. As demonstrated by studies published since the consensus guidelines, future observational studies with large sample sizes and randomized controlled trials are needed to determine if trough-guided vancomycin dosing and monitoring remain clinically relevant in specific patient populations. Maintaining a vancomycin trough level of < 15 mg/L may continue to be a viable option, particularly among patients with non-severe clinical status, but may not be appropriate for patients with severe infections.

## Data Availability

The datasets used and/or analyzed during the current study are available from the corresponding author on reasonable request.

## Abbreviations

AKI: Acute Kidney Injury
AUC: Area Under Curve
ICU: Intensive Care Unit
IDSA: The Infectious Diseases Society of America
MRSA: Methicillin Resistant Streptococcus Aures
MICs: Minimal inhibitory concentrations
VIN: Vancomycin Induced Nephrotoxicity

## References

[1] Rudd KE, Johnson SC, Agesa KM, Shackelford KA, Tsoi D, Kievlan DR, Colombara DV, Ikuta KS, Kissoon N, Finfer S, Fleischmann-Struzek C, Machado FR, Reinhart KK, Rowan K, Seymour CW, Watson RS, West TE, Marinho F, Hay SI, Lozano R, Lopez AD, Angus DC, Murray CJL, Naghavi M. Global, regional, and national sepsis incidence and mortality, 1990–2017: analysis for the global burden of Disease Study. Lancet. 2020;395:200–11. 10.1016/S0140-6736(19)32989-7. 31954465 10.1016/S0140-6736(19)32989-7 PMC6970225

[2] Rhee C, Dantes R, Epstein L, Murphy DJ, Seymour CW, Iwashyna TJ, Kadri SS, Angus DC, Danner RL, Fiore AE, Jernigan JA, Martin GS, Septimus E, Warren DK, Karcz A, Chan C, Menchaca JT, Wang R, Gruber S, Klompas M, Program CDCPE. Incidence and Trends of Sepsis in US hospitals using clinical vs Claims Data, 2009–2014. JAMA. 2017;318:1241–9. 10.1001/jama.2017.13836. 28903154 10.1001/jama.2017.13836 PMC5710396

[3] Rhee C, Jones TM, Hamad Y, Pande A, Varon J, O’Brien C, Anderson DJ, Warren DK, Dantes RB, Epstein L, Klompas M, Centers for Disease C, Prevention Prevention Epicenters P. Prevalence, underlying causes, and Preventability of Sepsis-Associated Mortality in US Acute Care hospitals. JAMA Netw Open. 2019;2:e187571. 10.1001/jamanetworkopen.2018.7571. 30768188 10.1001/jamanetworkopen.2018.7571 PMC6484603

[4] Li Y, Guo J, Yang H, Li H, Shen Y, Zhang D. Comparison of culture-negative and culture-positive sepsis or septic shock: a systematic review and meta-analysis. Crit Care. 2021;25:167. 10.1186/s13054-021-03592-8. 33964934 10.1186/s13054-021-03592-8 PMC8106121

[5] Phua J, Ngerng W, See K, Tay C, Kiong T, Lim H, Chew M, Yip H, Tan A, Khalizah H, Capistrano R, Lee K, Mukhopadhyay A. Characteristics and outcomes of culture-negative versus culture-positive severe sepsis. Crit Care. 2013;17:R202. 10.1186/cc12896. 24028771 10.1186/cc12896 PMC4057416

[6] Kim JS, Kim YJ, Kim WY. Characteristics and clinical outcomes of culture-negative and culture-positive septic shock: a single-center retrospective cohort study. Crit Care. 2021;25:11. 10.1186/s13054-020-03421-4. 33407768 10.1186/s13054-020-03421-4 PMC7787242

[7] Evans L, Rhodes A, Alhazzani W, Antonelli M, Coopersmith CM, French C, Machado FR, McIntyre L, Ostermann M, Prescott HC, Schorr C, Simpson S, Wiersinga WJ, Alshamsi F, Angus DC, Arabi Y, Azevedo L, Beale R, Beilman G, Belley-Cote E, Burry L, Cecconi M, Centofanti J, Coz Yataco A, De Waele J, Dellinger RP, Doi K, Du B, Estenssoro E, Ferrer R, Gomersall C, Hodgson C, Moller MH, Iwashyna T, Jacob S, Kleinpell R, Klompas M, Koh Y, Kumar A, Kwizera A, Lobo S, Masur H, McGloughlin S, Mehta S, Mehta Y, Mer M, Nunnally M, Oczkowski S, Osborn T, Papathanassoglou E, Perner A, Puskarich M, Roberts J, Schweickert W, Seckel M, Sevransky J, Sprung CL, Welte T, Zimmerman J, Levy M. Surviving sepsis campaign: international guidelines for management of sepsis and septic shock 2021. Intensive Care Med. 2021;47:1181–247. 10.1007/s00134-021-06506-y. 34599691 10.1007/s00134-021-06506-y PMC8486643

[8] Chong YP, Park SJ, Kim HS, Kim ES, Kim MN, Park KH, Kim SH, Lee SO, Choi SH, Jeong JY, Woo JH, Kim YS. Persistent Staphylococcus aureus bacteremia: a prospective analysis of risk factors, outcomes, and microbiologic and genotypic characteristics of isolates. Med (Baltim). 2013;92:98–108. 10.1097/MD.0b013e318289ff1e. 10.1097/MD.0b013e318289ff1e PMC4553980 23429353

[9] Ok HS, Lee HS, Park MJ, Kim KH, Kim BK, Wi YM, Kim JM. Predictors and clinical outcomes of persistent methicillin-resistant Staphylococcus aureus bacteremia: a prospective observational study. Korean J Intern Med. 2013;28:678–86. 10.3904/kjim.2013.28.6.678. 24307843 10.3904/kjim.2013.28.6.678 PMC3846993

[10] Wi YM, Rhee JY, Kang CI, Chung DR, Song JH, Peck KR. Clinical predictors of methicillin-resistance and their impact on mortality associated with Staphylococcus aureus bacteraemia. Epidemiol Infect. 2018;146:1326–36. 10.1017/S0950268818001255. 29781425 10.1017/S0950268818001255 PMC9134285

[11] Steinmetz T, Eliakim-Raz N, Goldberg E, Leibovici L, Yahav D. Association of Vancomycin serum concentrations with efficacy in patients with MRSA infections: a systematic review and meta-analysis. Clin Microbiol Infect. 2015;21:665–73. 10.1016/j.cmi.2015.04.003. 25887712 10.1016/j.cmi.2015.04.003

[12] Liu C, Bayer A, Cosgrove SE, Daum RS, Fridkin SK, Gorwitz RJ, Kaplan SL, Karchmer AW, Levine DP, Murray BE, M JR, Talan DA, Chambers HF. Infectious diseases Society of A. clinical practice guidelines by the infectious diseases society of America for the treatment of methicillin-resistant Staphylococcus aureus infections in adults and children. Clin Infect Dis. 2011;52:e18–55. 10.1093/cid/ciq146. 21208910 10.1093/cid/ciq146

[13] Bruniera FR, Ferreira FM, Saviolli LR, Bacci MR, Feder D, da Luz Goncalves Pedreira M, Sorgini Peterlini MA, Azzalis LA, Campos Junqueira VB, Fonseca FL. The use of Vancomycin with its therapeutic and adverse effects: a review. Eur Rev Med Pharmacol Sci. 2015;19:694–700. 25753888

[14] Aljohani S, Layqah L, Masuadi E, Al Alwan B, Baharoon W, Gramish J, Baharoon S. Occurrence of Vancomycin MIC creep in methicillin resistant isolates in Saudi Arabia. J Infect Public Health. 2020;13:1576–9. 10.1016/j.jiph.2020.07.008. 32859551 10.1016/j.jiph.2020.07.008

[15] Fujimori T, Hagiya H, Iio K, Higashionna T, Kakehi A, Okura M, Minabe H, Yokoyama Y, Otsuka F, Higashikage A. Vancomycin MIC creep progresses in methicillin-resistant Staphylococcus aureus despite the national antimicrobial stewardship campaign: single facility data in Japan. J Infect Chemother. 2022;28:918–22. 10.1016/j.jiac.2022.03.017. 35351391 10.1016/j.jiac.2022.03.017

[16] Arshad F, Saleem S, Jahan S, Tahir R. Assessment of Vancomycin MIC Creep Phenomenon in Methicillin-Resistant Staphylococcus aureus isolates in a Tertiary Care Hospital of Lahore. Pak J Med Sci. 2020;36:1505–10. 10.12669/pjms.36.7.3273. 33235565 10.12669/pjms.36.7.3273 PMC7674903

[17] Sader HS, Fey PD, Limaye AP, Madinger N, Pankey G, Rahal J, Rybak MJ, Snydman DR, Steed LL, Waites K, Jones RN. Evaluation of Vancomycin and daptomycin potency trends (MIC creep) against methicillin-resistant Staphylococcus aureus isolates collected in nine U.S. medical centers from 2002 to 2006. Antimicrob Agents Chemother. 2009;53:4127–32. 10.1128/AAC.00616-09. 19635961 10.1128/AAC.00616-09 PMC2764224

[18] Diaz R, Afreixo V, Ramalheira E, Rodrigues C, Gago B. Evaluation of Vancomycin MIC creep in methicillin-resistant Staphylococcus aureus infections-a systematic review and meta-analysis. Clin Microbiol Infect. 2018;24:97–104. 10.1016/j.cmi.2017.06.017. 28648858 10.1016/j.cmi.2017.06.017

[19] Joana S, Pedro P, Elsa G, Filomena M. Is Vancomycin MIC creep a worldwide phenomenon? Assessment of S. Aureus Vancomycin MIC in a tertiary university hospital. BMC Res Notes. 2013;6: 65. 10.1186/1756-0500-6-65. 23422012 10.1186/1756-0500-6-65 PMC3585458

[20] Pea F, Viale P, Furlanut M. Antimicrobial therapy in critically ill patients: a review of pathophysiological conditions responsible for altered disposition and pharmacokinetic variability. Clin Pharmacokinet. 2005;44:1009–34. 10.2165/00003088-200544100-00002. 16176116 10.2165/00003088-200544100-00002

[21] Drennan PG, Begg EJ, Gardiner SJ, Kirkpatrick CMJ, Chambers ST. The dosing and monitoring of Vancomycin: what is the best way forward? Int J Antimicrob Agents. 2019;53:401–7. 10.1016/j.ijantimicag.2018.12.014. 30599240 10.1016/j.ijantimicag.2018.12.014

[22] Murphy JE, Gillespie DE, Bateman CV. Predictability of Vancomycin trough concentrations using seven approaches for estimating pharmacokinetic parameters. Am J Health Syst Pharm. 2006;63:2365–70. 10.2146/ajhp060047. 17106010 10.2146/ajhp060047

[23] Rybak M, Lomaestro B, Rotschafer JC, Moellering R Jr, Craig W, Billeter M, Dalovisio JR, Levine DP. Therapeutic monitoring of vancomycin in adult patients: a consensus review of the American Society of Health-System Pharmacists, the Infectious Diseases Society of America, and the Society of Infectious Diseases Pharmacists. Am J Health Syst Pharm. 2009;66:82–98. 10.2146/ajhp080434. 19106348 10.2146/ajhp080434

[24] Holmes NE. Using AUC/MIC to guide Vancomycin dosing: ready for prime time? Clin Microbiol Infect. 2020;26:406–8. 10.1016/j.cmi.2019.12.023. 31927116 10.1016/j.cmi.2019.12.023

[25] Pritchard L, Baker C, Leggett J, Sehdev P, Brown A, Bayley KB. Increasing Vancomycin serum trough concentrations and incidence of nephrotoxicity. Am J Med. 2010;123:1143–9. 10.1016/j.amjmed.2010.07.025. 21183005 10.1016/j.amjmed.2010.07.025

[26] Elyasi S, Khalili H, Dashti-Khavidaki S, Mohammadpour A. Vancomycin-induced nephrotoxicity: mechanism, incidence, risk factors and special populations. A literature review. Eur J Clin Pharmacol. 2012;68:1243–55. 10.1007/s00228-012-1259-9. 22411630 10.1007/s00228-012-1259-9

[27] Filippone EJ, Kraft WK, Farber JL. The nephrotoxicity of Vancomycin. Clin Pharmacol Ther. 2017;102:459–69. 10.1002/cpt.726. 28474732 10.1002/cpt.726 PMC5579760

[28] Lodise TP, Patel N, Lomaestro BM, Rodvold KA, Drusano GL. Relationship between initial Vancomycin concentration-time profile and nephrotoxicity among hospitalized patients. Clin Infect Dis. 2009;49:507–14. 10.1086/600884. 19586413 10.1086/600884

[29] Prybylski JP. Vancomycin Trough Concentration as a predictor of clinical outcomes in patients with Staphylococcus aureus Bacteremia: a Meta-analysis of Observational studies. Pharmacotherapy. 2015;35:889–98. 10.1002/phar.1638. 26497475 10.1002/phar.1638

[30] Barriere SL, Stryjewski ME, Corey GR, Genter FC, Rubinstein E. Effect of Vancomycin serum trough levels on outcomes in patients with nosocomial pneumonia due to Staphylococcus aureus: a retrospective, post hoc, subgroup analysis of the phase 3 ATTAIN studies. BMC Infect Dis. 2014;14:183. 10.1186/1471-2334-14-183. 24708675 10.1186/1471-2334-14-183 PMC4101862

[31] Horey A, Mergenhagen KA, Mattappallil A. The relationship of nephrotoxicity to Vancomycin trough serum concentrations in a veteran’s population: a retrospective analysis. Ann Pharmacother. 2012;46:1477–83. 10.1345/aph.1R158. 23073306 10.1345/aph.1R158

[32] Rybak MJ, Le J, Lodise TP, Levine DP, Bradley JS, Liu C, Mueller BA, Pai MP, Wong-Beringer A, Rotschafer JC, Rodvold KA, Maples HD, Lomaestro BM. Therapeutic monitoring of Vancomycin for serious methicillin-resistant Staphylococcus aureus infections: a revised consensus guideline and review by the American Society of Health-System Pharmacists, the Infectious Diseases Society of America, the Pediatric Infectious Diseases Society, and the Society of Infectious diseases pharmacists. Am J Health Syst Pharm. 2020;77:835–64. 10.1093/ajhp/zxaa036. 32191793 10.1093/ajhp/zxaa036

[33] Folkers A, Anderson R, Harris J, Rogen C. The safety and efficacy of AUC/MIC-Guided vs trough-guided Vancomycin Monitoring among veterans. Fed Pract. 2023;40:28–33. 10.12788/fp.0346. 37223240 10.12788/fp.0346 PMC10201943

[34] McClure S, McElroy L, Gugkaeva Z. Implementation of Vancomycin AUC/MIC dosing vs traditional trough dosing and incidence of acute kidney injury at a rural community hospital. Am J Health Syst Pharm. 2024;81:e283–8. 10.1093/ajhp/zxae014. 38253056 10.1093/ajhp/zxae014

[35] Morales Junior R, Tiguman GMB, D’Amaro Juodinis V, Santos I, Leite FS, Vercelino JG, de Lima BD, Barbosa LMG. Trough-guided versus AUC/MIC-guided vancomycin monitoring: a cost analysis. Clin Ther. 2022;44:e91–6. 10.1016/j.clinthera.2022.07.012. 36031477 10.1016/j.clinthera.2022.07.012

[36] Page MJ, McKenzie JE, Bossuyt PM, Boutron I, Hoffmann TC, Mulrow CD, Shamseer L, Tetzlaff JM, Akl EA, Brennan SE, Chou R, Glanville J, Grimshaw JM, Hrobjartsson A, Lalu MM, Li T, Loder EW, Mayo-Wilson E, McDonald S, McGuinness LA, Stewart LA, Thomas J, Tricco AC, Welch VA, Whiting P, Moher D. The PRISMA 2020 statement: an updated guideline for reporting systematic reviews. BMJ. 2021;372: n71. 10.1136/bmj.n71. 33782057 10.1136/bmj.n71 PMC8005924

[37] Moher D, Liberati A, Tetzlaff J, Altman DG, Group P. Preferred reporting items for systematic reviews and meta-analyses: the PRISMA statement. PLoS Med. 2009;6: e1000097. 10.1371/journal.pmed.1000097. 19621072 10.1371/journal.pmed.1000097 PMC2707599

[38] Stang A. Critical evaluation of the Newcastle-Ottawa scale for the assessment of the quality of nonrandomized studies in meta-analyses. Eur J Epidemiol. 2010;25:603–5. 10.1007/s10654-010-9491-z. 20652370 10.1007/s10654-010-9491-z

[39] Higgins JP, Jackson D, Barrett JK, Lu G, Ades AE, White IR. Consistency and inconsistency in network meta-analysis: concepts and models for multi-arm studies. Res Synth Methods. 2012;3:98–110. 10.1002/jrsm.1044. 26062084 10.1002/jrsm.1044 PMC4433772

[40] Higgins JP, Thompson SG. Quantifying heterogeneity in a meta-analysis. Stat Med. 2002;21:1539–58. 10.1002/sim.1186. 12111919 10.1002/sim.1186

[41] Egger M, Davey Smith G, Schneider M, Minder C. Bias in meta-analysis detected by a simple, graphical test. BMJ. 1997;315:629–34. 10.1136/bmj.315.7109.629. 9310563 10.1136/bmj.315.7109.629 PMC2127453

[42] Jeffres MN, Isakow W, Doherty JA, McKinnon PS, Ritchie DJ, Micek ST, Kollef MH. Predictors of mortality for methicillin-resistant Staphylococcus aureus health-care-associated pneumonia: specific evaluation of Vancomycin pharmacokinetic indices. Chest. 2006;130:947–55. 10.1378/chest.130.4.947. 17035423 10.1378/chest.130.4.947

[43] Hermsen ED, Hanson M, Sankaranarayanan J, Stoner JA, Florescu MC, Rupp ME. Clinical outcomes and nephrotoxicity associated with Vancomycin trough concentrations during treatment of deep-seated infections. Expert Opin Drug Saf. 2010;9:9–14. 10.1517/14740330903413514. 20021290 10.1517/14740330903413514

[44] Kullar R, Davis SL, Levine DP, Rybak MJ. Impact of Vancomycin exposure on outcomes in patients with methicillin-resistant Staphylococcus aureus bacteremia: support for consensus guidelines suggested targets. Clin Infect Dis. 2011;52:975–81. 10.1093/cid/cir124. 21460309 10.1093/cid/cir124

[45] Clemens EC, Chan JD, Lynch JB, Dellit TH. Relationships between Vancomycin minimum inhibitory concentration, dosing strategies, and outcomes in methicillin-resistant Staphylococcus aureus bacteremia. Diagn Microbiol Infect Dis. 2011;71:408–14. 10.1016/j.diagmicrobio.2011.08.002. 21924852 10.1016/j.diagmicrobio.2011.08.002

[46] Hou Y, Ren J, Li J, Jin X, Gao Y, Li R, Zhang J, Wang X, Li X, Wang G. Relationship between mean vancomycin trough concentration and mortality in critically ill patients: a multicenter retrospective study. Front Pharmacol. 2021;12: 690157. 10.3389/fphar.2021.690157. 34349650 10.3389/fphar.2021.690157 PMC8326564

[47] Wang Y, Dai N, Wei W, Jiang C. Outcomes and nephrotoxicity associated with vancomycin treatment in patients 80 years and older. Clin Interv Aging. 2021;16:1023–35. 10.2147/CIA.S308878. 34103905 10.2147/CIA.S308878 PMC8179733

[48] Huang M, Wu H, Zhou J, Xu M, Zhou S. Efficacy of vancomycin on gram-positive bacterial infection in elderly critical patients and risk factors associated with nephrotoxicity. Arch Iran Med. 2018;21:349–55. 30113856

[49] Chuma M, Makishima M, Imai T, Tochikura N, Suzuki S, Kuwana T, Sawada N, Komatsu T, Sakaue T, Kikuchi N, Yoshida Y, Kinoshita K. Relationship between initial vancomycin trough levels and early-onset vancomycin-associated nephrotoxicity in critically ill patients. Ther Drug Monit. 2018;40:109–14. 10.1097/FTD.0000000000000459. 29095798 10.1097/FTD.0000000000000459

[50] Yahav D, Abbas M, Nassar L, Ghrayeb A, Kurnik D, Shepshelovich D, Leibovici L, Paul M. The association of Vancomycin trough levels with outcomes among patients with methicillin-resistant Staphylococcus aureus (MRSA) infections: retrospective cohort study. PLoS ONE. 2019;14: e0214309. 10.1371/journal.pone.0214309. 30946754 10.1371/journal.pone.0214309 PMC6448937

[51] Arasteh O, Khalili H, Taghi Beigmohammadi M, Abdollahi A, Mohammadpour A, Salehi M. Correlation between serum vancomycin trough level and therapeutic response in septic patients during augmented renal clearance phase. Arch Anesth Crit Care. 2019;5. 10.18502/aacc.v5i3.1203.

[52] Kralovicova K, Spanik S, Halko J, Netriova J, Studena-Mrazova M, Novotny J, Grausova S, Koren P, Krupova I, Demitrovicova A, Kukuckova E, Krcmery V Jr. Do Vancomycin serum levels predict failures of Vancomycin therapy or nephrotoxicity in cancer patients? J Chemother. 1997;9:420–6. 10.1179/joc.1997.9.6.420. 9491842 10.1179/joc.1997.9.6.420

[53] Bosso JA, Nappi J, Rudisill C, Wellein M, Bookstaver PB, Swindler J, Mauldin PD. Relationship between Vancomycin trough concentrations and nephrotoxicity: a prospective multicenter trial. Antimicrob Agents Chemother. 2011;55:5475–9. 10.1128/AAC.00168-11. 21947388 10.1128/AAC.00168-11 PMC3232787

[54] Chung J, Oh JM, Cho EM, Jang HJ, Hong SB, Lim CM, Koh YS. Optimal dose of Vancomycin for treating methicillin-resistant Staphylococcus aureus pneumonia in critically ill patients. Anaesth Intensive Care. 2011;39:1030–7. 10.1177/0310057X1103900608. 22165354 10.1177/0310057X1103900608

[55] Arshad S, Shoyinka A, Chen A, Jacobsen G, Zervos M. Evaluation of Vancomycin serum trough concentrations and outcomes in meticillin-resistant Staphylococcus aureus bacteraemia. Int J Antimicrob Agents. 2012;40:474–5. 10.1016/j.ijantimicag.2012.06.020. 22883416 10.1016/j.ijantimicag.2012.06.020

[56] Tongsai S, Koomanachai P. The safety and efficacy of high versus low Vancomycin trough levels in the treatment of patients with infections caused by methicillin-resistant Staphylococcus aureus: a meta-analysis. BMC Res Notes. 2016;9:455. 10.1186/s13104-016-2252-7. 27686168 10.1186/s13104-016-2252-7 PMC5041442

[57] Meng L, Fang Y, Chen Y, Zhu H, Long R. High versus low Vancomycin serum trough regimen for Gram-positive infections: a meta-analysis. J Chemother. 2015;27:213–20. 10.1179/1973947814Y.0000000182. 24641266 10.1179/1973947814Y.0000000182

[58] Hale CM, Seabury RW, Steele JM, Darko W, Miller CD. Are vancomycin trough concentrations of 15 to 20 mg/L Associated with increased attainment of an AUC/MIC >/= 400 in patients with presumed MRSA infection? J Pharm Pract. 2017;30:329–35. 10.1177/0897190016642692. 27074786 10.1177/0897190016642692

[59] Lodise TP, Lomaestro B, Graves J, Drusano GL. Larger Vancomycin doses (at least four grams per day) are associated with an increased incidence of nephrotoxicity. Antimicrob Agents Chemother. 2008;52:1330–6. 10.1128/AAC.01602-07. 18227177 10.1128/AAC.01602-07 PMC2292536

[60] Jeffres MN, Isakow W, Doherty JA, Micek ST, Kollef MH. A retrospective analysis of possible renal toxicity associated with Vancomycin in patients with health care-associated methicillin-resistant Staphylococcus aureus pneumonia. Clin Ther. 2007;29:1107–15. 10.1016/j.clinthera.2007.06.014. 17692725 10.1016/j.clinthera.2007.06.014

[61] van Hal SJ, Paterson DL, Lodise TP. Systematic review and meta-analysis of Vancomycin-induced nephrotoxicity associated with dosing schedules that maintain troughs between 15 and 20 milligrams per liter. Antimicrob Agents Chemother. 2013;57:734–44. 10.1128/AAC.01568-12. 23165462 10.1128/AAC.01568-12 PMC3553731

[62] Pan C, Wen A, Li X, Li D, Zhang Y, Liao Y, Ren Y, Shen S. Development and validation of a risk prediction model of Vancomycin-Associated Nephrotoxicity in Elderly patients: a pilot study. Clin Transl Sci. 2020;13:491–7. 10.1111/cts.12731. 31785129 10.1111/cts.12731 PMC7214653

