# Correction: Effect of low vs. high Vancomycin trough level on the clinical outcomes of adult patients with sepsis or gram-positive bacterial infections: a systematic review and meta-analysis

**DOI:** 10.1186/s12879-025-10745-5

**Published:** 2025-03-17

**Authors:** Subhash Chander, Roopa Kumari, Hong Yu Wang, Yaqub Nadeem Mohammed, Om Parkash, Sindhu Lohana, FNU Sorath, Abhi Chand Lohana, FNU Sadarat, Sheena Shiwlani

**Affiliations:** 1 Department of Medicine, Icahn School of Medicine, Mount Sinai, New York, NY USA; 2 Department of Medicine, Montefore Medical Center, Bronx, NY USA; 3 Department of Medicine, AGA khan University Hospital, Karachi, Pakistan; 4 Department of Medicine, Dow University Health Sciences, Karachi, Pakistan; 5 Department of Medicine, Western Michigan University, Kalamazoo, WV USA; 6 Department of Medicine, University at Bufalo, Bufalo, NY USA; 7 Department of Pathology, Icahn School of Medicine, Mount Sinai, New York, NY USA; 8 Mount Sinai Beth Israel Hospital, 281 1st Ave, New York, NY 10003 USA

Following publication of the original article [1], we were notified that the last sentence in the Results section in the Abstract was incorrect.

Originally published sentence:

However, low trough levels were associated with a non-signifcant trend towards a lower risk of treatment failure [OR = 0.89 (95% CI 0.73–1.10), *p* = 0.28] and were signifcantly associated with reduced risk of all-cause mortality [OR = 0.74 (95% CI 0.62–0.90), *p* = 0.002].

Corrected sentence:

However, lower trough levels were significantly associated with reduced microbial clearance [OR = 0.47 (95% CI 0.23–0.96), *p* = 0.04]. Additionally, low trough levels showed a non-significant trend toward a lower risk of treatment failure [OR = 0.89 (95% CI 0.73–1.10), *p* = 0.28] but were significantly associated with a reduced risk of all-cause mortality [OR = 0.74 (95% CI 0.62–0.90), *p* = 0.002].

The original article has been corrected.
